# Awakenings in triplicate: in ontogeny, after immobility, and to the presence of a congener. Comments on Ilan Golani’s concept of the mobility gradient

**DOI:** 10.3389/fnbeh.2024.1325481

**Published:** 2024-05-30

**Authors:** Koenraad Kortmulder

**Affiliations:** Department of Biology, Leiden University, Leiden, Netherlands

**Keywords:** awakening, ontogeny, immobility, locomotion, vertebrates, behavioral expansion, social behavior, congener

## Abstract

Golani’s concept of the “mobility gradient” describes the unfolding of motor behavior after immobility and in ontogeny. The two processes run parallel and are similar across vertebrates. In both time scales, the trend is for the behavior to progressively become enriched, cover more dimensions (motor expansion) and become less dependent on external stimuli (stimulus-bound). This paper addresses the question whether the gradient extends into social, interactive behavior. Observation of natural groups larger than dyads may help answering that question. As an example, the natural social behavior of a fish, *Pethia nigrofasciata* is described in some detail. It is concluded that their motor behavior expands in the course of their daily spawning period.

## Introduction: a discussion of the “mobility gradient”

Ilan Golani’s experiments describe in great detail, the movements of vertebrate animals that awaken from natural or induced sleep (Golani, 1992). His subjects include mammals, reptiles, birds, and amphibians. The order in which the elements of the behavior appear, as it expands from immobility to the full locomotor pattern, is surprisingly similar, not only between conspecifics, but also across vertebrates and, to some extent, even between them and arthropods. Golani (1992) coined the term “mobility gradient” for the whole process. First come horizontal movements, second forward, third vertical against a support, fourth unsupported vertical, all relative to the hind legs.^1^ Each of these “modules” develops gradually in antero-posterior (head-to-tail) direction. One by one, the modules increase the freedom of movement of the animal (motor expansion). Concurrently, dependency on external beacons (stimulus-bound) decreases. The reverse process (motor constriction) occurs under stress or under the influence of certain drugs.

Even more surprising is that this entire pattern repeats, in an accelerated form, the first activation of the same responses in the individual’s ontogeny. Golani (2022) points to the parallel between these antero-posterior progressions and the embryonic development of the body. In its turn, the longitudinal order of body segments is collinear with a series of Hox genes, situated on each of four chromosomes. Each Hox gene is associated with a certain range of embryonic segments (Golani, 2022). It is tempting to think of a causal relationship, in which the Hox genes determine the order of the developing body segments, and play a role also in the behavioral development.

Golani’s experiments with single animals are very convincing. If the “mobility gradient” extends into social behavior, it would represent an underlying order that has so far be neglected in motivation analyses. Locomotion, after all, lies at the base of many behaviors, including social behavior. Grazing, for instance, demands slow locomotion to be coordinated with the feeding acts; mating behavior should bring together the genitals. Hunting requires a variety of locomotor rhythms (sneaking up to a prey, jumping on it). The choreography of contest and play implicate perfect coordination of stepping depending on what the partner does (Golani, 1976, 2022). Play requires gesturing “this is play” (Bateson, 1956), and other signals must accompany exchanges of soft touch (*tederheid*, *teder* behavior, Kortmulder, 1994, 2021; Dubbeldam and Kortmulder, 2009) Would the same orders of elements persist in these contexts? Or could new requirements have selected for other patterns? What is the evidence?

Golani and co-workers have arranged social encounters between conspecifics, mostly mammals, such as jackals, wolves, honey badgers, rats, and Tasmanian devils (Golani, 2022). In my view, such two-by-two interactions, in which struggling for dominance plays a main role, are of limited value for tracing the “mobility gradient.” That is because, in these encounters, the *sequences* of elements—essential for the theory—may be disturbed by stress in both interactants. If either expansion or constriction may reign, the sequences observed are difficult to interpret. Another disturbing factor are “oppositions”: the prolonged contact or proximity of certain body parts of the two animals while other parts keep moving. For example, the snout-to-cheek opposition of honey badgers. During an opposition the steps of both partners may still be influenced by the “mobility gradient” (Golani, 2022), but oppositions at least delay the unfolding of motor expansion (or constriction).

However, indirect evidence was extracted from the observations by comparing the behaviors of relatively inferior partners with dominant ones. The former were typically limited to the “earlier,” more restricted movements of the gradient, while the latter had the whole spectrum at their disposal (Golani, 1992).

As to birds, Golani takes his evidence from the literature. Vertical movements (in the median sagittal plane) predominate in the courtship of most species. Exceptions are mannikins and birds-of-paradise, in which male courtship displays are performed in various other planes. Also here, much remains uncertain; for instance, it is not yet clear whether in birds-of-paradise the vertical displays *precede* the more complicated ones in time, as would be expected on the basis of the mobility gradient model (Golani, 2022).^2^

**Figure 1:**
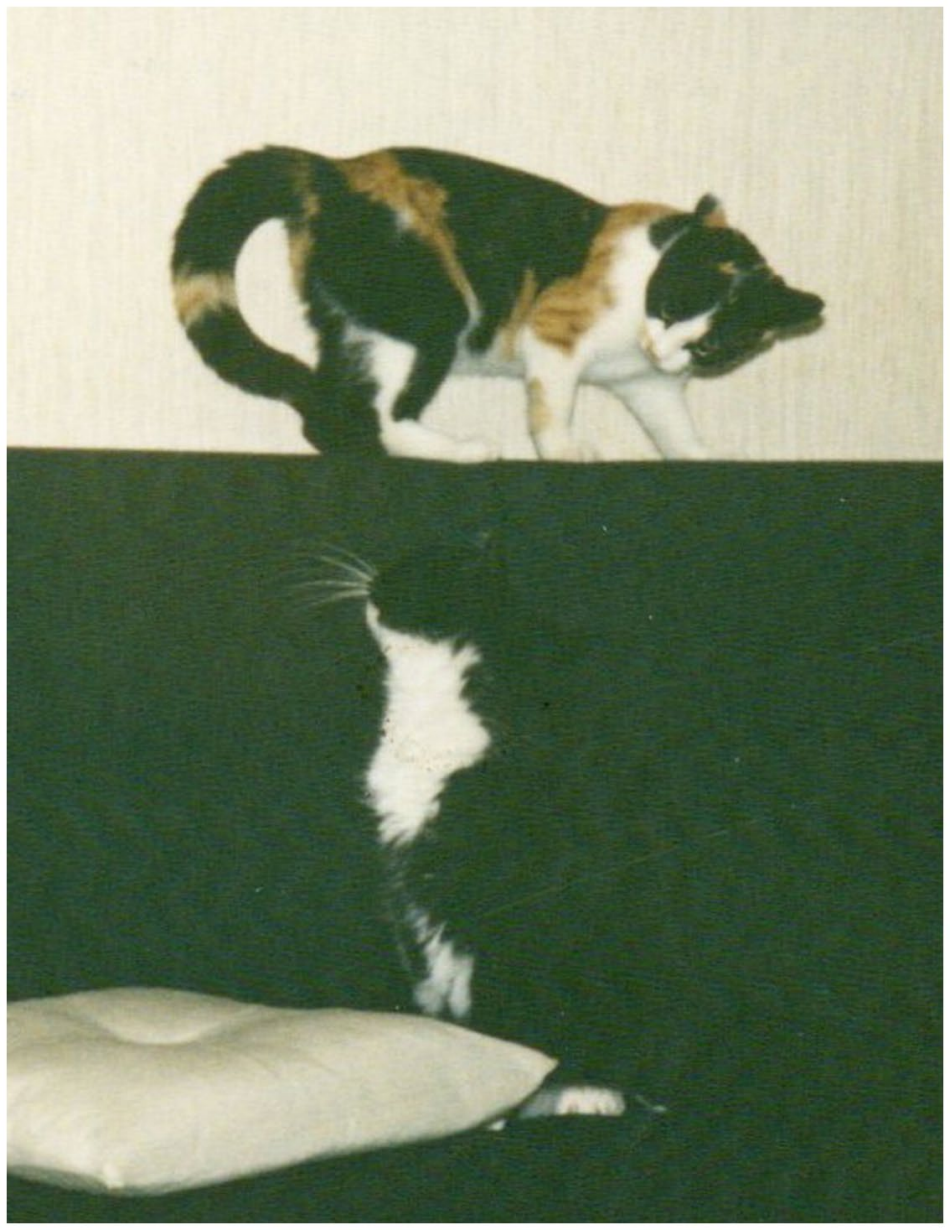
The signal “this is play” as given by a cat (top one in the picture).

Additional evidence may be gleaned from incipient play behavior and its signaling “this is play” in dogs and cats: lowering the body on the forelegs in dogs (play bow) or the elevated hindquarters plus S-bend in the tail of cats (Figure 1). While these signals are mainly constrained to the vertical plane, the playful interaction that may follow moves more freely through several planes and rotations.

Golani also mentions that the play-fighting social behavior of developing wolf cubs keeps pace with the growth of their mobility gradient as tested individually. This strongly suggests a direct role of the gradient in social interaction (Golani, 1992).

## Awakening to the presence of a congener

The concept of behavioral expansion was first proposed by Kortmulder (1974, 1983). It had a slightly different accent from Golani’s concept of motor expansion, in that the former related to social behavior only. In the early 1970’s, I began to realize that there was something in common in the courtship behaviors of fish species as different as Cyprinid barbs, sticklebacks, diverse cichlids, guppies, and Anabantids, and indeed of many birds as well. Despite all the differences between their species-specific “dances”, on meeting a conspecific of the right sex, they all displayed a burst of activity encompassing some behaviors not usually seen in the isolated individual. One aspect already generally known was the occurrence of fragments from various behavioral contexts, such as aggressive, fearful, parental, nest-building behavior, or preening. Various analyses had yielded diverse interpretations of their appearance such as ambivalences, displacement activities, and vegetative responses. Yet, irrespective of such details, there was the increased activity and variation in all those species, and I needed a name for the phenomenon: I called it behavioral expansion. A similar, though less varied, storm of activity followed meetings between same-sex individuals, particularly males. Soon, I had to distinguish motorial vs. sensorial expansion, because of differences between for instance the playful courtship of male barbs with the “feeling” courtship of Anabantids. Next, I realized that even more than courtship in fishes and birds, play behavior of birds and mammals (largely motor) and *tederheid* (mainly sensory) displayed a diversity not seen in normal behavior (Kortmulder, 1983). Play can function as courtship in dogs, *tederheid* (grooming) has a role in courtship in monkeys, and both play and *tederheid* may function similarly in humans.^3^

When I first mentioned the question in a paper (1974), I emphasized (p. 72) that behavioral expansion was a new notion, derived from observation, but not referring to any known underlying principle. That was before I knew Ilan Golani’s work on the genesis of motor behavior! Since then, his experimental work has provided a staunch underpinning to the concept.

## Larger groups

Observations on two interacting individuals in a laboratory setting allow great precision and detail. Larger, more natural groups, however, may offer more scope for longer series of motor expansion. In order to show that the trend of motor expansion extends into social behavior, such groups may thus be preferred. As an example, I offer a description of the full social behavior of a Cyprinid fish, *Pethia nigrofasciata*.^4^ There are multiple reasons for my choosing this species. First, fishes are under-represented in Golani’s experiments, though his theories are about Vertebrates at large. Second, *Pethia nigrofasciata* deploys its full social (spawning) behavior in daily periods of a few hours, beginning from scratch each early morning, and ending around midday. Third, it happens to be one of a whole series of related species with the behavior of which I am very familiar through almost life-long studies both in the aquarium and in the field.

Two behavior elements performed among males may be described first: the Lateral Display (*alias* Lateral Threat) and Dorsal/Ventral Roll. The Lateral Display is widely spread among Cypriniform, Characiform, and other orders of fishes. It is part of the agonistic behavior, particularly of and among males. The display may be described as follows: the body is held rigid, probably through contraction of antagonistic muscles, notably those that lie at either side of the spine. The fins are held stiffly erected. The body may be drawn into a tight, lateral C- or S-curve, while the tail may move in a regular rhythm as in fast swimming. Waggling (pivoting around a vertical axis through the center of the body) is another common component, similar to tail-beating in other fish. All through, the performer tends to turn its side to another fish, even to the extent of rolling over if the other is above or below (a case of *opposition* in Golani’s terminology). This orientation component makes the body and fin colors of the performer maximally visible to the addressee. Besides its being performed in one place, the display may be accompanied by fast forward locomotion, often of an apparently “compulsive” nature because of awkward steering. When two males are engaged in mutual lateral display, they may get trapped together into a duel culminating in fast spinning at close quarters around a common vertical axis (the so-called carousel). From this “trap,” they can only escape by either one giving up and becoming inferior. On and off with the display, the body, and fins color black; the most intense coloring is seen when a male is engaged in fighting *and* courting in a spawning group.

Lateral display is an expression of self-confidence. A barb in lateral display will stand its “ground” against a conspecific attacker and may induce the latter to retreat or to display in return (Dunham et al., 1968). A submissive barb, on the other hand, performs Dorsal or Ventral Rolling that is: it abandons all tensions in the body muscles and firmly adduces all fins to the body. Instead of its side, it rolls the narrow surface of its back or belly toward the stronger fish (another *opposition*). It maintains that orientation to the other one irrespective of their relative positions. In the natural interaction between males, dorsal/ventral rolling expresses the willingness to break off contact. It makes the performer look small and non-threatening, and accordingly the aggressor loses interest (Dunham et al., 1968).

Male courtship consists of an array of circling and leading moves centered around a female, the whole is performed in a continuous, quivering locomotion with adducted fins and variable leaning toward the female. It must be noted that “leaning” is not an opposition. When the male is below (and to the side of) the female, the male’s dorsal edge is more or less turned toward the female, but when the male is above (and to the side of) her, his lateral surface is more visible to her than the ventral and dorsal edges. In other words, the male’s angle of leaning does not depend on his relative position to the female. At irregular intervals (Putters et al., 1984) during the dance, male and female perform brief mating clasps in each of which they shed a few eggs and some sperm. In a group, mating is promiscuous. There is no pause following a mating; courtship is immediately resumed. Whenever males meet males, they may display laterally or roll the back or belly toward the rival. A courting male may “keep-off” others from the courted female, normally with lateral display toward the intruder, or he may briefly attack the latter from keeping-off position. Such rare males as defend territories, give them up as soon as courtship reaches the stage of mating. In a group, the great majority of the males are colored black with a red head, whereas females maintain the normal pattern of black cross-bars on a light ground. Prior to courtship, when females are still unwilling, males attack and display to each other in increasing measure, starting at dawn, while their spawning colors begin to develop.

To understand its significance, the reproductive behavior must be observed in the species’ natural habitat, small rivers in the hills of South-West Sri Lanka. There they meet in spawning groups, daily and throughout the year during the later morning hours, at specific locations that are characterized by relatively shallow water, abated current, and sunshine. Together such places form roughly 2% of the river’s surface (Kortmulder et al., 1978). We observed spawning particularly in three such locations with 20–150 fishes for approximately 16 h in total, and never saw duels between males reaching the stage of carousel, nor any signs of territories. The spawning unfolds similarly in the field as in the laboratory: first increasing fighting and displaying of males, next the courtship phase culminating about an hour after its onset. As in the aquarium, pauses in the willingness of the females are filled by males returning temporarily to the fight- and display phase. The whole begins soon after sunrise, when fish of both sexes begin to appear in the spawning ground. A few hours later, they depart one by one, back to the main river, where feeding takes place.

In the laboratory, even in large tanks of 130 cm × 65 cm ground surface (six males six females), male duels proceeding to carousel occur now and then, increasing in frequency in smaller tanks with fewer individuals. In groups of two males and two females in 60 cm × 35 cm tanks, continued dominance of one male over the other was normal, except, temporarily, in the case that both females were willing at the same time. In all-male groups of two or three, dominance of one was the rule (Kortmulder, 1972). Apparently, containment causes stress, and a narrowing down of the freedom and variability of behavior (motor constriction). In *Pethia nigrofasciata*, the natural situation represents the most expanded form of its reproductive behavior, and this fish species can get very close to the communal play of higher vertebrates. Generalizing over vertebrates, behavioral constriction through stress invariably leads to agonistic behavior ending up in a permanent dominant-submission condition, unless the loser can escape.

Thus, the full reproductive behavior of *P. nigrofasciata* unfolds in time: from rest, via lateral display among males to courtship which culminates in frequent mating. Whereas male–male displaying occurs in a single plane—that plane may be tilted relative to the real horizontal, but carousels are always horizontal—courting fish move freely through three dimensions. At the height of activity, the occurrence of the *oppositions* between males (Lateral Display and Ventral/Dorsal Roll) is reduced to brief moments, except when two or more males pursue a female. The only remaining stimulus binding the behavior is the mating clasp. The behavior thus unfolds similarly to the behavior of other vertebrates, namely from relatively simple to more complex, and *vice-versa* under stress (Golani, 1992, 2022).

But where does it all begin? Not with immobility, at least not in the natural situation. In the aquarium, however, I often induced immobility by transporting a fish from one tank to another. In its new surroundings, such a fish may for a while lie still, only breathing, upon the bottom of the tank and/or between plants. As I have had neither fish nor facilities for two decades, I must rely on memory. The first movement made by an immobile fish is a vigorous forward one with visible yaw of the head. In one jerk, this movement frees it from the bottom and plants to swim around the tank. Hence, at least theoretically, it could join in spawning behavior of those already present. Are we witnessing, in those cases, the homolog of the horizontal movements of higher vertebrates after immobility?

Here, I might warn against an idealization of natural behavior. The spawning groups of *Pethia nigrofasciata*, without territories, represent a limit case, shared only with few other species of related Cyprinids. Most barb species spawn in territories and exhibit much more stress also in nature (Kortmulder, 1972, 1982; Kortmulder and Robbers, 2017).

An interesting question at this point is why animals should go to the length and trouble of play-like behavior in order to reach mating. I suggest that it is because behavioral expansion loosens the grip that agonistic motivations (and other ego-functions in the Freudian sense) tend to have on the normal (non-reproductive) behavior, and thus allows uniting with another individual and the release of the animal’s own gametes.

## Motorial vs. sensorial aspects of behavior

Golani studied mainly the motorial aspect of behavior. Perhaps similar orders may be found in the tactile sensitivity of animals. I noticed that dogs as well as cats, when willing to be stroked, first offer the head, next the neck and the back in that order, only much later followed (if at all) by the ventral surface and the extremities. An interesting case to be followed up consists in the preening after bathing in birds. The only published example that I know is van Iersel and Bol’s (1958) study of preening in two tern species. Their interpretations are in terms of drives and threshold values—as usual at the time. The order in which the diverse parts are preened may suggest a progress from simpler to more complicated movements. New data, involving many more species of birds will be easy to collect.

## The congener

Along with the notion of behavioral expansion came the concept of congener, because “another animal” is the natural stimulus eliciting the former (Kortmulder, 1974, 1986). “Congener,” rather than “conspecific” because, particularly in play and to some extent in *tederheid*, other species may be accepted partners. The concept of congener implies a cognitive image that is present in most animals but can be damaged by early experience or certain genes. It refers to the ability of an animal (or human) to compare the behavior of another animal with its own behavior. It thus relates to the presence of mirror neurons in mammals and some birds, but I presume that similar functions are already exerted by other parts of the brain, in lower vertebrates at least.

The concept is somewhat similar to Lorenz (1935), in that it may refer only to a particular sort of conspecific at a time; but it also allows for “wider than the own species,” namely in play, where other species may be accepted as partner. (As to *tederheid*: animals of many species readily let themselves be stroked at least by humans). The cognitive image of “who is a congener” thus seems to widen and narrow with the expansion and narrowing of visible behavior (Kortmulder, 1986).

## Data availability statement

The original contributions presented in the study are included in the article, further inquiries can be directed to the corresponding author.

## Ethics statement

Ethical approval was not required for the study involving animals in accordance with the local legislation and institutional requirements because was not in vigor during the years of this study (far before 2000).

## Author contributions

KK: Conceptualization, Formal Analysis, Investigation, Methodology, Visualization, Writing – original draft.

## Footnotes

^1^Two more maneuvers follow later, in the context of fighting or playing: horizontal, respectively, vertical with weight on forelegs.

^2^One may wonder why these examples of courtship begin with movements in the vertical plane, and not with the horizontal movements as characteristic of the mobility gradient. It must be noted; however, that courtship does not usually start from immobility (Golani, pers. comm.). The starting positions of males in the pair-forming aggregations of wild mallards may indeed look like immobility, but it is only the above-water parts of the males that look relaxed. All the time, these males are jockeying for positions where their displays will be seen to full advantage by the most attractive female.

^3^Besides motor and sensory expansion, I distinguished “motivational expansion,” to account for the appearance in courtship of whole fragments of behavior elements known to belong to other motivations.

^4^Perhaps better known as *Puntius nigrofasciatus* or Black Ruby. Pictures of a male of the species may be found at www.aquainfo.nl/artikel./pethia-nigrofasciata-purperkopbarbeel/. The male on the left is relaxing from lateral display, the black body and fin colors still lingering, the fins already returning to neutral positions. In full display, the leading edges of the dorsal, ventral and anal fins are perpendicular to the body outline.
